# Quality of life and trust among young people with narcolepsy and their families, after the Pandemrix® vaccination: protocol for a case-control study

**DOI:** 10.1186/s12887-017-0935-4

**Published:** 2017-08-23

**Authors:** Karin Blomberg, Agneta Anderzén Carlsson, Lars Hagberg, Östen Jonsson, Lena Leissner, Mats H. Eriksson

**Affiliations:** 1 Faculty of Medicine and Health, School of Health Sciences, Örebro University, S-70182 Örebro, Sweden; 2 Faculty of Health, Science and Technology, Department of Health Sciences, Karlstad University, Karlstad, Sweden; 3 Faculty of Medicine and Health, University Health Care Research Centre, Örebro University, Örebro, Sweden; 4 Faculty of Medicine and Health, Department of Paediatrics, Örebro University, Örebro, Sweden; 5 Faculty of Medicine and Health, Department of Neurology, Örebro University, Örebro, Sweden

**Keywords:** Life change events, Mass vaccination, Narcolepsy, Trust, Quality of life

## Abstract

**Background:**

The extensive vaccination programme against swine flu resulted in an increased incidence of narcolepsy among children and adolescents. There is a need to explore if these young persons’ experiences have affected their trust in healthcare, their willingness to participate in future prevention programmes, and their contacts with the healthcare system. The overall aim is to identify factors important for the life-situation of children and adolescents with narcolepsy and their families, and factors that correlate with trust in healthcare.

**Methods/design:**

Data will be collected via questionnaires from all available children with narcolepsy following the vaccination and their families, as well as a control group of children with diabetes and their families. Longitudinal descriptive interviews will also be conducted with a selection of 20–25 children and their families. Techniques from media research will be used for Internet-based data collection and analysis of information relating to narcolepsy from social media.

**Discussion:**

This project will use the situation of young persons with narcolepsy after the swine flu vaccination as a case to build a model that can be used in situations where trust in healthcare is essential. This model will be based on findings from the included studies on how trust is influenced by support, quality of life, burden of disease, impact on family, and use of social media. The model developed in this project will be beneficial in future situations where trust in healthcare is essential, such as new pandemic outbreaks but also for “everyday” adherence to health advice.

## Background

### Narcolepsy as a consequence of Pandemrix® vaccination

In 2010, an increased number of newly diagnosed narcolepsy cases among children and adolescents were seen, as a consequence of the comprehensive national vaccination campaign with Pandemrix® against H1N1-influenza (the swine flu) that took place during winter 2009–10. Studies recently published in Sweden, Finland, UK and Ireland have demonstrated that there is a link between narcolepsy and the Pandemrix® vaccine [1] with the latter producing an increase in the risk of narcolepsy [2, 3]. For Sweden, it means approximately 350 children and adolescents who acquired narcolepsy after vaccination against swine flu [4]. The long-term consequences for this group of young persons is not yet known, but a doubling of personal and economic burden and healthcare consumption has been shown in an adult narcolepsy population [5].

### The role of health-literacy and trust in the healthcare system

The fact that so many young persons and their families were affected by this condition as a consequence of their trust in an official health information campaign has many similarities to other events such as the thalidomide-tragedy of the 1960s, impairing young persons’ life-situations but also leading to a debate about health information [6]. Similar questions about future difficulties with obtaining trust arose after the swine flu vaccination campaign and are likely to do so again in the future as a pattern of increased global mobility increases the risk of new pandemic outbreaks [7].

There are several potential risks, from both a societal and individual perspective, if only a part of the population agrees to be immunized against a potential pandemic pathogen. A vaccine program has to cover the majority of the population in order to limit an outbreak, but it is also an effective way of protecting individuals who for different reasons cannot be vaccinated or who are immunosuppressed. To make a decision about vaccination is described as being a complex process. In a re-interpretation of reasons for not attending the mass vaccination against swine flu in Sweden, several personal experiences together with stories by media are described to have influenced the decision making [8]. Attitudes to vaccinations are formed by both experiences and health literacy; that is, ability to understand, synthesize and apply information regarding to health. Health literacy is seen as an important factor for the individual’s health outcome [9], and especially for improving young persons and their families’ health and healthcare utilization [10]. However, today there is limited evidence about the mechanisms affecting the health literacy within a population [10], and there are no existing models that can be used to enhance health information and thus improve both health literacy and adherence to health recommendations such as vaccination programmes. The homogeneity of the group of young persons with narcolepsy makes them a possible case for elaborating on such a model, since this population is well-identified and similar in age, time of symptom debut and perceived cause of the disease.

In a study of reasons for people not to take part in swine flu vaccination, mistrust emerged as one of the explanations [11], although there are no studies into the degree and significance of confidence among people who took part in vaccination and who were later diagnosed with narcolepsy. Similar phenomena of mistrust can be found in other fields relating to vaccination, such as vaccination against human papillomavirus (HPV) [12, 13] and measles [14]. We have previously revealed a lack of confidence in society in young women’s reasoning regarding the HPV-vaccine [15, 16]. The link between narcolepsy and the campaign for vaccination against swine flu is often cited as an example of why it is impossible to rely on medical expertise [17]. Researchers have proposed that the trust which paediatric patients and their parents place in physicians and other health professionals affects the patients’ adherence to medical regimes and, as a consequence, their physical health, and quality of life [18–20]. Trust can be defined by Rotenberg’s Basis, Domain, and Dimension (BDT) framework with three bases (*reliability, emotional trust,* and *honesty),* two domains (*cognitive/affective* and *behavioural*), and two target dimensions (*specificity* and *familiarity*) which together create a framework of interpersonal trust [21]. To study the experiences of children and adolescents who are suffering from post-vaccine narcolepsy can gain knowledge of whether this has affected their and their families’ trust in healthcare system. Such knowledge can be used to prepare and be more proactive if/when similar situations with a need of mass-vaccination might occur.

### The role of internet and social media

Despite a lack of systematic and scientific documentation concerning the wellbeing and quality of life of these children/adolescents and their families, there are numerous descriptions in social media of how life has been affected since the vaccination, as well as the difficulty involved in acquiring help and support from health and medical care services [22, 23].Various voluntary actions have been initiated and described in social media by people affected, or by people close to them.

Communication is an important part of handling a situation where an individual or family member is affected by a serious and/or chronic disease, and historically this has been dealt with in personal meetings. The development of the media and altered communication patterns, primarily among young people, means that different types of media and the Internet have a major role to play in providing support for life events and crises, and can also influence personal perceptions [24]. Social media allows the individual to find other people affected by the same condition, irrespective of geographical distance [25, 26]. However, studies have found that when it comes to other crises, such as having a parent suffering from cancer, use of the Internet for information and support was of minor importance [27]. Nevertheless, it is important to gain an increased understanding and knowledge of the significance of and opportunities for support provided by various forms of social media such as online blogs and other, more interactive forums for children with narcolepsy following swine flu vaccination, and for their families. Social media are often used to amplify opinions rather than as a forum for “objective” discussion [24, 28, 29]. The rapid dissemination of opinions and the central role assigned to the Internet as a means of gathering information present a challenge to the healthcare services in that they have to deal with rapid changes in opinions of medical knowledge, and sometimes even misleading information published by laymen.

### The LISAN-project

The project is run by the research group LISAN (Life-situation for Children and Adolescents with Narcolepsy) at Örebro University and Region Örebro County. The group members have an extensive, unique expertise from clinical experience of chronically ill children (including children with narcolepsy), knowledge of the relevant scientific methods, and research among children and adolescents. LISAN works in cooperation with an advisory board of national and international experts in the field of narcolepsy and with stakeholders from patient organisations.

The project is guided by the expectation that the affected children/adolescents and their families may show lower trust beliefs in physicians, as well as in other health professionals (i.e. nurses), and therefore be at higher risk for no adherence to prescribed medical regimes, poorer health, and lower quality of life than other children (i.e., the comparison group). Therefore, the overall aim of the project is to identify factors important for the life-situation of children and adolescents with narcolepsy and their families, and factors that correlate to trust in healthcare. To do this we will examine the wellbeing and quality of life of the children and their families; the impact of narcolepsy on daily life, including going to school, leisure time, and relationships with friends; the need for support expressed by the children and their families; the significance of social media; the health and economic consequences for the children and their families and economic consequences for society; the trust in healthcare itself. The knowledge gained in the project can be used contribute in future situations where trust in healthcare is essential, such as new pandemic outbreaks, but also for “everyday” adherence to health advice.

## Methods and design

The project involves descriptive and exploratory studies with cross-sectional, prospective, and retrospective design and both qualitative and quantitative methodology. Data will be collected via questionnaires, including both existing relevant and valid instruments and newly developed and validated such, administered to all available children with narcolepsy following the vaccination and their families, as well as to a control group of children with diabetes and their families. Furthermore, registry data of earning among parents and health care use will be utilized and longitudinal, descriptive interviews involving a selection of 20–25 children with narcolepsy following vaccination with Pandemrix® and their families will be conducted. Techniques from media research will be used for Internet-based data collection and analysis of information relating to narcolepsy from social media. Finally, data and findings from all the preceding studies will be used for analyses of trust, adherence, and health-economic aspects.

### Sample and recruitment

In October 2012, a total of 205 cases of narcolepsy following vaccination with Pandemrix® were recorded in the Swedish adverse event database, of which 173 were children [30]. New cases are still being diagnosed [31] and a recent report suggest that approximately 350 children and adolescents were affected by narcolepsy after vaccination against swine flu [4]. The children included must be old enough to be able to understand and express how they perceive their wellbeing and quality of life. To allow for cultural and ethnic diversity, professional interpreters will be used for contact with families who do not speak Swedish. “Family” refers to people who live with the child, such as parents, step-parents and siblings.

All identified Swedish cases of children (age < 18 years at disease onset) with narcolepsy (regardless of types of narcolepsy), following vaccination with Pandemrix® will be invited to the study. We will select cases i.e. those who have a Narcolepsy diagnosis documented in the Swedish nationally register of diagnosis (diagnose code G47.4). No exclusion criteria will be used.

Children with diabetes mellitus type 1 will serve as comparison group. The control group has been selected to represent a condition that impacts daily life, possibly for the entire family, and increases the need for support. Selection for the control group will take place via the patient register, with selections based on matched controls (i.e. equivalent children with diabetes: same number, similar ages, same sex).

A purposeful sampling procedure will be used for the qualitative data collection, involving approximately 20–25 children with narcolepsy following vaccination with Pandemrix®, based on achieving a variation of sex, age, socio-economic status, culture, and geographical location.

### Outcomes and procedures

A longitudinal, prospective comprehensive survey of all children in the narcolepsy and diabetes groups (and their families) will be carried out in respect of wellbeing and quality of life. The survey instruments have been selected to capture the factors important to the wellbeing and quality of life of children and their families, the impact of narcolepsy on daily life, the need for support expressed by the children and their families, the significance of social media, health economic consequences of the condition and finally; the trust in healthcare. These outcomes will be compared between respondents, over time, and with children and adolescents with other chronic conditions (i.e. diabetes). Most of the children will be old enough to respond to the instruments. Specific adaptation of the instruments to the narcolepsy population will be performed where necessary. Demographic and medical data (such as medications and other diseases) and information on care contacts will be collected from the adverse event and narcolepsy registers [30, 32] and from children’s medical journals. Data from the quality of life instruments will be used for estimation of burden of disease. The survey will be repeated after two years in order to capture any change in the parameters studied.

#### Instruments

A majority of the instruments already exist in validated Swedish language versions (see below). For the remaining instruments the research group has initiated an extensive work translating and validating them, in accordance with the ISPOR guidelines [33].

##### Health-related quality of life

SF-36 [34] is a general quality of life instrument which includes 36 questions providing an 8-scale profile of functional health and wellbeing scores, as well as psychometrically-based physical and mental health summary measures and a preference-based index. The scales included are: physical functioning, role-physical, bodily pain, general health, vitality, social functioning, role-emotional and mental health. The instrument will be answered by the parents.

##### Quality of life among children with chronic conditions

DISABKIDS Chronic Generic Measure (DCGM-37) is a general quality of life survey standardised for 8–18-year old European children with chronic conditions [35]. The results consist of a total score and six dimensions: independence (managing on their own, feeling positive), emotion, social inclusion, social exclusion, physical limitations and medication.

Younger children (aged 4–7) will instead be asked to respond to a version known as DISABKIDS Smileys Take 6 [36]. Children with narcolepsy or diabetes will respond to DCGM/DISABKIDS Smileys.

KIDSCREEN [37] is a 27-item quality of life-instrument for children and adolescents aged 8–18 years. The result is divided into five dimensions: physical wellbeing, psychological wellbeing, autonomy & parents’ relation, peers & social support, and school environment.

PROMIS [38] is a new quality of life instrument presently being developed and validated for paediatric use in Sweden. It is adaptive, meaning that the answer on one question leads to the next question, thus optimizing the data collection.

##### Activity

The Children’s Assessment of Participation and Enjoyment/Preferences for Activities of Children (CAPE/PAC) [39] is a 55-item instrument for children and young people aged 6–21 years, measuring participation in and enjoyment of free-time activities.

##### Trust in, and adherence to, health and medical care services

Children’s Trust Beliefs in General Physicians (CTBGP) [21] is a 9-item instrument that assesses children’s trust belief in physicians. The Multidimensional Trust in Health Care System Scale (MTHCSS) [40] includes 17 questions for measuring confidence in caregivers and health and medical care institutions. In addition, questions about adherence to medical regimes, adapted from works by Rotenberg [21] and Hayford [41], will be asked.

##### Impact on the family

The Quality of Life in a Child’s Chronic Disease Questionnaire (QLCCDQ) [42] was developed in order to examine how the family is affected by a child’s chronic condition. This instrument consists of 15 questions within six domains: family roles, social roles, occupational roles, role limitations, symptoms and emotions. Parents and siblings over the age of 15 will respond to QLCCDQ.

##### Impact on alertness

The Epworth Sleepiness Scale (ESS) [43], which was used in earlier narcolepsy studies [44], is a list of eight situations (e.g. watching TV, sitting and talking to someone) where the person asked has to specify how great the risk is of them falling asleep. Children with narcolepsy will respond to the ESS.

The Ullalinna Narcolepsy Scale (UNS) [45] is a 12-item narcolepsy-scale previously used in narcolepsy-research.

##### Social media

A social network analysis tool [46] will be utilised in order to collect data and chart how specific search terms related to narcolepsy following vaccination occur in social media. This tool will allow quantitative data to be obtained relating to the number of users, communication patterns (what people talked about, who talked), temporal and geographical aspects of usage, dissemination of information, links between social media activity and other events such as news reports in traditional media and types of discussions (emotional or factual). The content of the communication will also be analysed regarding issues as impact of narcolepsy on daily life, need for support and trust in healthcare.

#### Registry data

##### Healthcare consumption

From national patient registries, data will be collected on inpatient care and physician visits in open specialist care. Unfortunately, register of primary care is lacking. Drug prescriptions will be retrieved from the Medical Register. Data will be collected for children with narcolepsy and their parents.

##### Earnings and production

All parents’ earnings will be followed from two years before the onset of narcolepsy and to the follow-up occasion. From the individual perspective, all earnings including salaries and contributions from society will considered, and from a societal perspective salaries (i.e. production) will be considered. Data will be collected from the national authority Statistics Sweden.

#### Qualitative outcomes

The selected children and their families will be interviewed on two occasions approximately two years apart. These interviews will focus on experiences and perceptions of the children and their families on wellbeing and quality of life; the impact of narcolepsy on daily life, including going to school, leisure time, and relationships with friends; the need for support expressed by the children and their families; the significance of social media; and trust in healthcare system. The interviews will follow the “laddered questions” interview technique [47]. Repeated interviews will also be carried out with parents, either individually or in pairs, and with siblings during the same period and with the same emphasis. Particular emphasis will be placed on how the child and their family describe their need for support from various bodies in society. The interviews will be conducted by researchers with experience of talking to children and adults and will take place in locations specified by the study participants themselves. Each interview is expected to take about an hour and a half.

### Data analysis

The interviews will be transcribed verbatim and analysed with qualitative content analysis [48], which is an inductive approach appropriate when knowledge of a field is limited. Data from the instruments will be analysed using descriptive and comparative statistical methods appropriate on the basis of the data level and distribution. Data processing and the analysis of quantitative data will take place in cooperation with statistical experts. Various computer-based tools for social network analysis [46] will be used to analyse social media. This method will make it possible to quantitatively analyse the number of users of various media, the frequency and amount of communication, time spent using media, patterns in dissemination of information, links between social media activity and specific events such as news reporting in traditional media and the nature of the communication (technical, emotional, factual, positive or negative, etc.). The significance of social media for these children and their families will be analysed in connection with the analysis of the interviews. Gender perspectives and gender differences will be taken into account when analysing data in all subprojects, for example with respect to differences in quality of life, roles and social media activity.

### Health economic consequences

The health economic consequences will be described and analysed in three parts. All will be compared to best available general population data: 1) the burden of disease, based on loss of quality of life expressed in DALYs (disability adjusted life years), 2) healthcare consumption of children and parents, and 3) the development of earnings and production for parents.

### Ethical considerations

The studies will be conducted in accordance with the Declaration of Helsinki and have already gained approval from an ethical review board (reg.no 2013/505). Participants will be given verbal and written information before being asked whether they would like to participate. The individual should have given consent that data from the registers may be used for research purposes before participation. For participants aged 15 and above, their own written informed consent will be considered sufficient, while for participants under 15, the consent of their parents will also be requested. Although children can be viewed as a group with a particular need for privacy protection in research, they also have the right to express their own opinions and have others listen to them, which creates an implicit need to initiate studies with children [49]. The members of the research team have extensive experience of talking to children in vulnerable situations, and are sensitive to any need for support. Structures will be established to ensure that children and parents can be referred on to an appropriate caregiver if contact with care services is needed. Data collected will be treated confidentially. Participation is voluntary, and participants will be entitled to withdraw from the study without having to give a reason.

### Knowledge dissemination

Results will be presented continuously during the project period as publications in scientific journals, as conference presentations, and in meetings with professionals to gauge the broader implications of the results. We will also share the results in reports to the participants, and invite patient organisations to a presentation of the study findings. To facilitate community engagement results will also be published in the daily press and social media.

## Discussion

It is well known that an impaired trust in healthcare leads to lower adherence to prescribed and preferred health behaviour in individuals which may be a serious risk factor for the general public. This project will use the situation of children and adolescents with narcolepsy after the swine flu vaccination as a case to build a model that can be used in situations where trust in healthcare is essential. The model will be based on findings from studies on how trust is influenced by support, quality of life, economic impact on family, and use of social media. The model developed in this project will be beneficial in future situations where trust in healthcare is essential, such as new pandemic outbreaks, but also for “everyday” adherence to health advice. Additionally, knowledge will be generated about the life-situation of the young persons with narcolepsy and their families. Without sufficient knowledge of these young persons’ quality of life and need for support, it is difficult to prevent any negative consequences for identity development, schooling, and future opportunities to lead a meaningful life, work, and health. In the long run, this knowledge can lead to better understanding of prevention in these children with a risk of developing psychosocial problems and other mental illnesses such as depression. Evaluating the consequences resulting from the condition will also create a foundation for target management and prioritisation in respect of distribution of resources and assessment of medical disability in the event of illness.

Additionally, the project will identify and permit the development of interventions relating to the daily lives of young persons and their families in order to promote their health and continued development. Knowledge will also be generated concerning the degree of impact on quality of life and whether the need for support differs from young persons with diabetes, which may be of significance to other patient groups. Dissemination of the results will be accelerated and facilitated by means of close cooperation with international experts in the field via the reference group and together with the patient association. Cooperation with the advisory board (experts and laymen) ensures that the studies carried out will be perceived as important and relevant from a social perspective.
